# Do Patients With Depression Prefer Literal or Metaphorical Expressions for Internal States? Evidence From Sentence Completion and Elicited Production

**DOI:** 10.3389/fpsyg.2018.01326

**Published:** 2018-08-15

**Authors:** Christina Kauschke, Nadine Mueller, Tilo Kircher, Arne Nagels

**Affiliations:** ^1^Clinical Linguistics, Department of German Linguistics, University of Marburg, Marburg, Germany; ^2^Department of Psychiatry and Psychotherapy, University of Marburg, Marburg, Germany; ^3^General Linguistics, Department of English and Linguistics, Johannes Gutenberg University Mainz, Mainz, Germany

**Keywords:** depression, internal state language, metaphor, figurative language, elicited speech production, formal thought disorder

## Abstract

In everyday communication metaphoric expressions are frequently used to refer to abstract concepts, such as feelings or mental states. Patients with depression are said to prefer literal over figurative language, i.e. they may show a concreteness bias. Given that both emotional functioning and the processing of figurative language may be altered in this clinical population, our study aims at investigating whether and how these dysfunctions are reflected in the understanding and production of metaphorical expressions for internal states. We used two behavioral approaches: a sentence completion task and elicited speech production. In the first experiment, patients with ICD 10 depression (*n* = 26) and healthy controls (*n* = 32) were asked to complete sentences by selecting an appropriate word out of four alternatives (metaphorical expression, literal expression, concrete distractor, abstract distractor). All participants–irrespective of the presence of depression–chose more literal (60%) than metaphorical (40%) expressions. In the second experiment, patients with depression (*n* = 44) and healthy controls (*n* = 36) described pictures showing emotive events. The descriptions were transcribed and coded for type of expression (non-figurative words for internal states vs. metaphorical expressions, valence, type of metaphor, source and target domain of metaphor). In addition, the Thought and Language Index was applied to assess formal thought disorder. When talking about internal states, both groups used more literal than metaphorical expressions. The groups did not differ with respect to the composition of internal state language, but patients with depression tended to verbalize positive content to a lesser extent. Correlation analyses within the patients' group revealed that signs of disorganization in their speech were related to a higher use of internal state expressions, whereas a negative correlation was found with dysregulation phenomena. Taken together, results indicate that people with and without depression prefer literal means in order to verbalize internal states, but they additionally make use of figurative language. Since patients with depression were able to understand and produce metaphors for internal states similar to controls, the concreteness bias cannot be confirmed by the present study. The results contribute to existing research by demonstrating associations between symptoms of formal thought disorder and internal state language.

## Introduction

The capacity of expressing one's own feelings and recognizing the feelings of others represents a key prerequisite for successful human communication. In everyday life situations, internal states, such as emotions, desires, intentions, perceptions, physiological sensations, or mental states, are verbally encoded by various means. These means are subsumed under the term “Internal State Language (ISL),” which can be expressed either in a literal/non-figurative or in a figurative way.

The vocabulary used to convey perceptions, emotions, and thought processes literally—the so-called “Internal State Terms (IST)” or “Psychological State Terms”—comprises lexical units that explicitly refer to concepts about internal states as symbols (Klann-Delius, 2015; Schwarz-Friesel, 2015). For example, emotion terms like “anger” or “joy” refer to specific affective states. Such words for internal states are acquired in a predictable developmental sequence “from relatively tangible physiological states to that of epistemic concepts” (Kristen et al., 2014, p. 6) that has been observed across languages: words for physiological states, sensory perception, and volition are followed by words for emotions, with words for moral judgements and cognition appearing latest (Bretherton and Beeghly, 1982; Kauschke and Klann-Delius, 1997; Kristen et al., 2012, 2014). While the early categories of IST are strongly related to bodily experience, emotion terms form the bridge to mental concepts. In this sense, emotion terms are a special category of words situated between concrete and abstract terms (Altarriba and Bauer, 2004). Their meaning is related to bodily symptoms, facial expressions, physiological reactions as well as to internal states of the organism. Therefore, they are considered to be a crucial stepping stone for the acquisition of abstract concepts, helping to grasp the distinction between entities existing in the physical world and those existing only in the human mind (Vigliocco et al., 2014). Because of the preponderance of affective information inherent in abstract words—and in particular in emotion terms—embodiment approaches have been applied to explain their acquisition and processing (Kousta et al., 2011). In addition to the highly relevant role of embodied affective experience, it has been frequently recognized that verbal context is crucial for learning and understanding abstract concepts. Linguistic information given in social situations plays a pivotal role for the expansion and differentiation of a vocabulary for internal states (Borghi et al. to appear, Vigliocco et al., 2014).

In clinical populations the use of IST may be altered compared to healthy subjects, as has been investigated exhaustively for neurodevelopmental disorders like autism spectrum disorder (ASD, e.g., Capps et al., 2000; Rumpf et al., 2012; Siller et al., 2014; Kauschke et al., 2015; Levy and Kauschke, 2015; see also the meta-analysis in Baixauli et al., 2016) or attention deficit hyperactivity disorder (ADHD, Miranda et al., 2013), but rarely for psychiatric disorders in adults.

While IST denote internal states literally, figurative means of verbalizing internal processes are also common in everyday communication: “It is a typical feature of affective language that it is highly figurative” (Schwarz-Friesel, 2015, p. 165). Figurative language encompasses various devices, most prominently metaphors. In the metaphorical expression “she is boiling” an angry person is described analogously to a container of hot fluid. Thus, an abstract concept (the affective state “anger”) is linked to a concrete, perceptible experience (concrete experience with hot fluids). In this sense, figurative expressions often evoke sensory-motor experiences that make the underlying concept more salient and accessible. In the cognitive metaphor theory of Lakoff and Johnson (1980/2002), Lakoff (2014) this conceptual mapping from a source domain (e.g., fluid in a container) to a target domain (e.g., anger) has been described in detail. In the same vein, Gibbs (2003) stresses that many source domains are rooted in sensorimotor experience. The presence of figurative constructions in language is therefore seen as support for the idea of an embodied grounding of linguistic meaning. Additionally, Foolen (2012) points out that figurative speech contributes to “involvement” in the sense that talking about feelings or inner states triggers emotional involvement. Figurative speech stimulates perceptible or somatic reactions that accompany internal states and thereby emphasizes the speaker's and/or hearer's involvement. In sum, there is corroborating evidence that the use of figurative expressions for internal states serves at least two functions:

- making abstract concepts and internal processes more concrete via embodiment and
- indicating involvement and expressivity.

Since the ability to understand and make use of figurative language may be altered in various disorders (Benítez-Burraco, 2017), clinical populations “offer a glimpse into subtle dissociations between literal and non-literal (figurative) language” (Vulchanova et al., 2015, p. 4). Dysfunctions in figurative language processing are commonly found in clinical populations such as ASD (e.g., Happé, 1995), Williams syndrome (Annaz et al., 2009), schizophrenia (e.g., Mossaheb et al., 2014), and major depression (Nagels et al., 2016). Moreover, an association was found between formal thought disorder (FTD) symptom severity in patients with depression and performance in a multiple-choice proverb test: a subcategorization into different FTD dimensions such as positive (e.g., derailment, crosstalk, etc.) and negative symptoms (e.g., poverty of thought, inhibited thinking, etc.) revealed that the negative dimension was related to proverb interpretation performance (Nagels et al., 2016). To date it is unclear how different FTD dimensions are associated to the use of internal state language in spontaneous speech.

The purpose of the present study is to learn how people use literal and metaphorical expressions for internal states and whether and how these processes/decisions are influenced in patients with mood disorder. In particular patients with depression seem to differ from healthy controls, since (a) dysfunctions in the processing of figurative language were reported, (b) they experience and express emotional involvement in a different way, and (c) they show symptoms of formal thought disorders. For this purpose, the processing and use of metaphorical expressions for internal states was investigated using two different behavioral tasks.

### Internal state language and figurative language in patients with depression

Patients suffering from major depression show characteristic symptoms, such as depressed mood, loss or diminishment of interest, pleasure, enjoyment or energy, and decreased activity (ICD-10). Anhedonia, a decreased sensitivity to pleasurable events, is considered as a major symptom of the disorder (Bevins and Besheer, 2005). Patients pay selective attention to negative stimuli, interpret stimuli more negatively than healthy controls, or show attenuated processing of positive stimuli. Such alterations in the processing of emotional stimuli have been shown for various kinds (e.g., facial expressions, music, prosody, sounds, Doose-Grünefeld et al., 2015). The following paragraphs will outline how the negative bias or other characteristics of the disorder are reflected in the patients' speech and language processing.

The ability to represent and structure inner experiences with language contributes positively to mental health. This connection between language and emotional experience is said to be reduced in most clinically depressed patients (Simşek, 2013). However, little is known about how individuals with depression verbalize their inner experiences, i.e., how they make use of ISL. It is thus largely unclear whether and how the amount and composition of ISL differ from those observed in healthy subjects. On the other hand, it is a well-known and frequently observed phenomenon that when talking about internal states, the negative bias is evident in patients' speech. Self-rumination, a strong focus on one's own problems and distress, has often been described as a typical symptom of depression (see Simşek, 2013; Zimmermann et al., 2017). As a consequence, language of depressed patients is generally characterized by a strong focus on the self and by an over-representation of negative content. In this vein, Rude et al. (2004) showed that depressed participants used more negative emotion words and produced the pronoun “I” more frequently as opposed to non-depressed participants. The latter finding seems to be also clinically relevant, as frequent first-person pronoun use was found to predict an unfavorable course of depression (Zimmermann et al., 2017). The negative bias is also reflected in the processing of verbal stimuli. Schlipf et al. (2013) demonstrated attenuated evaluation of positive information in patients with depression for both verbal and non-verbal emotions. Using a lexical decision task, Stip et al. (1994) found that patients with depression were particularly slow in processing affective words. Lexical decision was also investigated by Canli et al. (2004), who found that depressed subjects exhibited less brain activation for happy words and more activation for sad words than did controls.

In particular in schizophrenia or mania it has been observed that abstracts ideas or concepts are interpreted in a concrete way, clinically referred to as concretism. Obviously, a lack of understanding and interpreting abstract information has implications for the successful processing of figurative language. Thus, idiomatic expressions and proverbs tend to be interpreted in a meaningless and concretistic way (Barth and Küfferle, 2001). To date, it is unclear whether concretism is also characteristic for depression. Barth and Küfferle (2001) state that concretistic interpretations of metaphors and proverbs in patients with depression are not as pronounced as in schizophrenia, but difficulties in the processing of figurative language cannot be fully excluded. Nagels et al. (2016) used the proverb interpretation test by Barth and Küfferle (2001) and showed that patients with depression had more difficulties interpreting metaphors and proverbs than healthy controls and therefore assume dysfunctions in abstract information processing. In a study by Iakimova et al. (2006) patients with depression and schizophrenia were asked to complete sentences by choosing a word with literal, figurative, concrete, or unrelated meaning. Participants with major depression and with schizophrenia showed a bias toward literal responses. These findings contrast with the results of a questionnaire study by Bartczak and Bokus (2015). Here, written sentences with differing degrees of metaphoricity were presented to the participants who were asked to indicate on a scale how well a sentence describes an abstract notion (e.g., future, sadness). Results showed that the ratings of literal and metaphorical sentences did not differ between subjects with depression and controls, since all participants preferred sentences with a moderate degree of metaphoricity. In the participants with depression, metaphorical processing was intensified for sentences with negative valence. The authors conclude that depressive subjects do not have problems with metaphorical processing *per se*, but demonstrate strong interpretational negativism. Taken together, it is still a topic of critical debate to what extent metaphorical processing is affected in patients with depression.

With regard to speech production, Charteris-Black (2012) analyzed how people with depression verbally express their attitude toward the disorder in oral interviews. Metaphors about the experience of depression frequently involved “trapped” feelings, “descent,” “weight and pressure,” and “darkness” as source domains. Similar types of metaphorical expressions have been found in McMullen and Conway (2002). Charteris-Black (2012) suggests that metaphorical expression of trapped feelings can facilitate the recovery process and recommends that therapists should encourage clients to use diverse metaphors to convey the intensity of their emotions. Levitt et al. (2000) describe in detail how the focused use of metaphors during therapy (“unloading the burden”) can even support recovery and contribute to a positive outcome. The authors therefore consider metaphors as a useful marker for psychotherapeutic change.

Given that emotional functioning and its expression in language as well as the processing and use of figurative language may be altered in patients with depression, the present study aims at investigating whether and how these dysfunctions are reflected a) in the understanding and b) in the production of literal and metaphorical expressions for internal states. We aim at answering the following questions:

- Do patients with depression differ from healthy controls with respect to their preference for literal or figurative expressions in a sentence completion task?
- Do patients with depression differ from healthy controls in the verbalization of internal states in elicited speech production? Differences may arise with respect to the number of internal state terms (IST), the valence of IST used, and the proportion of literal vs. figurative expressions of internal states.
- Are individual differences in the presence of positive or negative formal thought disorder (FTD) related to the verbalization of internal states?

## Experiment 1—sentence completion

### Participants

This study included 26 patients diagnosed with depression (10 male, 16 females, mean age 37.26 years, SD 10.59, range 33 years). They were recruited and tested at the Department of Psychiatry and Psychotherapy, Marburg. All patients were diagnosed with depression by experienced psychiatrists according to ICD-10 criteria and received anti-depressive medication and/or mood stabilizers. The Beck Depression Inventory (Beck, 1961) was used to assess the current severity of the patients' depression. According to BDI classification criteria, 17% of the patients were in remitted states of depression, 21% displayed mild symptoms of depression, 38% showed moderate depression, and 25% showed severe symptoms.

As a control group, 32 healthy participants (12 male, 20 females, mean age 37.47, SD 11,91, range 37 years) were recruited through postings and local advertisements. Sixty-five percent of the participants in both groups had a higher education level (a-level equivalent). All participants were native speakers of German, gave written informed consent, and were paid EUR 5 for their participation. The study was approved by the local ethics committee of the Medical Faculty.

### Stimuli

A paper pencil sentence completion task was designed comprising 23 written verbal contexts. After a short sentence introducing the context, a second, incomplete sentence was presented which had do be completed by one of four alternative choices:

1. adequate figurative completion of the sentence
2. adequate literal completion of the sentence
3. near semantic distractor
4. unrelated distractor.

The four alternatives were presented in randomized order throughout the experiment and covered various emotional, mental, physiological, or internal states.

One example from the German item set is:

*Sie stieg in das fremde Auto* (she entered the stranger's car). *Das war* (this was) …

1. *blauäugig* (starry-eyed, metaphorical for naïve)
2. *naiv* (naïve)
3. *wachsam* (watchful)
4. *primitiv* (primitive)

The introductory sentences plus the incomplete target sentences had a mean length of 11.13 (*SD* = 1.18) syllables and included a balanced proportion of female and male personal pronouns. Word frequencies of all endings were obtained from a German frequency data base (see http://wortschatz.uni-leipzig.de). Between the four response options (see above) mean frequencies did not differ significantly. With respect to the valence of the whole construction (introductory sentence, target sentence, and completion), 12 constructions were positive or neutral, and 11 were negative.

### Procedure

All participants completed a test of proverb and metaphor interpretation (Barth and Küfferle, 2001). This multiple-choice test contains fourteen metaphorical sayings. Participants were asked to choose the correct proverb interpretation among five response alternatives presented in random order. The response alternatives represent different degrees of severity of concretistic thinking. Afterwards, the sentence completion experiment was conducted. Participants were instructed to read the sentences carefully and to tick the response alternative that appeared most appropriate for them. There was no time constraint on the task.

### Data analysis

The proportion of each response type was calculated for each participant. The response patterns were compared between the groups using non-parametric group comparisons, one subject-based (Mann-Whitney-U-Test) and one item-based (Wilcoxon-Test). In addition, correlations were calculated between the scores obtained from the proverb and metaphor interpretation test and the performance in the sentence completion task.

### Results

Regarding the test of proverb and metaphor interpretation (Barth and Küfferle, 2001), patients with depression scored significantly lower as compared to healthy controls with respect to the number of correct interpretations (Healthy Controls: *M* = 13.16; *SD* = 1.82; Patients with Depression: *M* = 11.54; *SD* = 2.99; *U* = −2.34, *p* < 0.02).

Table 1 illustrates that participants with and without depression showed a similar response pattern in the sentence completion task. Patients with depression chose significantly more distractors, however, the proportion of distractors was very low throughout the experiment (see Table 1). Importantly, across the experiment both groups selected figurative endings at around 40% and the literal completion at nearly 60%. The proportion of figurative and literal endings did not differ between groups (see Table 1). In addition, no correlations were found between performance in the proverb interpretation test and the proportion of literal or figurative endings. However, the proportion of near semantic distractors was significantly associated with the proverb interpretation test performance (Spearman's *rho* = 0.367, *p* > 0.05) in the patient group.

**Table 1:** Response patterns in the sentence completion task.

		**Figurative ending**		**Literal ending**		**Near distractor**		**Unrelated distractor**	
		**Subject based**	**Item based**	**Subject based**	**Item based**	**Subject based**	**Item based**	**Subject based**	**Item based**
Healthy controls	Mean SD	41.4% 10.8	41.6% 26.3	58.0% 10.6	57.5% 26.5	0.5% 1.8	0.5% 1.5	0%	0%
Patients with depression	Mean SD	41.5% 13.37	38.4% 23.3	56.5% 14.3	59.5% 23.8	1.5% 3.2	1.4% 2.5	0.8% 1.9	0.8% 2.1
Group comparison		*U* = −0.502 ns	*Z* = −1.217 ns	*U* = −0.517 ns	*Z* = −0.684 ns	*U* = −1.527 ns	*Z* = −2.297 *p* = 0.022	*U* = −2.278 *p* = 0.023	*Z* = −1.890 *p* = 0.059

*Means and standard deviations for the percentages of each response type are reported for subject- and item-based analyses*.

### Summary of experiment 1

The sentence completion experiment did not reveal any differences between groups: All participants—irrespective of diagnosis—chose more literal (60%) than metaphorical (40%) expressions in order to complement sentences conveying internal states. Our data show that literal expressions are preferred over figurative ones, but figurative expressions were also evaluated as appropriate, although to a lesser degree. The results from this task do not suggest dysfunctions in the processing of figurative language in depression. This finding is in line with the results of Bartczak and Bokus (2015) who also found that metaphor processing in patients with depression was similar to that of healthy controls. Having shown that patients with depression are likewise able to understand and interpret figurative expressions in context, the next question is whether they make use of this option in their spontaneous or elicited speech production.

## Experiment 2—elicited speech production

### Participants

This study included 44 patients diagnosed with depression (27 male, 17 females, mean age 45.5 years, SD 14.9, range 56 years). They were recruited and tested at the Department of Psychiatry and Psychotherapy, Marburg. All patients were diagnosed with depression by experienced psychiatrists according to ICD-10 criteria and received anti-depressive medication and/or mood stabilizers. The Hamilton Depression Scale (HAM-D) was used to assess depressive symptom severity. According to HAM-D criteria, 20% of the patients were remitted, 27% displayed mild symptoms of depression, 39% showed moderate depression, and 14% showed severe depression.

A group of 36 healthy control subjects (16 male, 20 females, mean age 36.4, SD 10.94, range 41 years) was recruited through postings and advertisements. Twenty-six percent of the patients with depression and 36% of the controls had a higher education level. All participants were native speakers of German, gave written informed consent, and were paid EUR 10 for their participation. The study was approved by the local ethics committee of the Medical Faculty.

### Stimuli

Speech production was elicited by the Thematic Apperception Test (TAT) developed during the 1930s (Murray, 1943). Participants are asked to describe ambiguous and emotionally laden black and white pictures of situations and people. For this purpose, these pictures are highly suitable for the elicitation of internal state language. For the present study, seven of the fourteen TAT-pictures were randomly chosen for each participant.

### Procedure

The proverb and metaphor interpretation test (Barth and Küfferle, 2001) was applied to all participants (s. description above).

Participants were asked to describe seven TAT-pictures within a given time window of 3 min for each picture. If necessary, the interviewer was allowed to encourage the ongoing speech production by asking non-suggestive questions (e.g., “Can you describe more details?” “Can you imagine what will happen next?”) (for further information on the procedure see Liddle et al., 2002). Each speech sample, comprising around 21 min of spontaneous speech per participant, was recorded and transcribed. Interviewers were blind to the diagnosis.

Formal thought disorder severity was assessed using the “Thought and Language Index” (TLI) (Liddle et al., 2002): disorganization (median = 0.5, min = 0 and max = 8), impoverishment (median = 2.7, min = 0 and max = 29.25) and non-specific dysregulation (median = 0.25, min = 0 and max = 4.5). This instrument was validated for the assessment of different FTD dimensions using the “Thematic Apperception Task” pictures.

### Data analysis

The transcripts were coded according to the following coding categories:

1. **Internal state terms:** Words that literally refer to internal states were coded according to a classification scheme based on Kauschke and Klann-Delius (1997) that has also been used in other studies (e.g., Rumpf et al., 2012; Miranda et al., 2013; Kauschke et al., 2015). The classification distinguishes between several kinds of internal states:
  - Physiology: terms for subjective, physical sensations (e.g. “tired”)
  - Terms for intention, desire, and obligation (e.g. “want”)
  - Emotion: terms for discrete emotions (e.g., “fear”), for facial emotional expressions (e.g., “smile”), and for bodily expressions of emotions such as movements or postures (e.g., “crouching”) (Wallbott, 1998)
  - Evaluation: terms that convey moral or emotional judgments
  - Cognition: terms for mental/cognitive states, expressions of knowledge, belief, remembrance.
    If participants used cognitive terms (like “I don't know”) in order to express their own uncertainty about the content of the picture, these utterances were separately coded as “hesitation phenomena” and excluded from the analyses. In addition, participants commented their perceptual act of looking at the picture extremely often. In order to avoid a distortion of the data, these utterances (containing verbs like “see,” “look,” “recognize”) were also excluded from the analyses.
    The valence of all IST was coded as positive, negative, or neutral.
2. **Figurative expressions for internal states**: All figurative expressions that conveyed internal states were analyzed. A coding system was developed that determined:
  - Type of metaphor (Lakoff and Johnson, 1980/2002; Kövecses and Benczes, 2010):
    - Container metaphor: metaphors in which emotional or physical states are represented as a container with boundaries (e.g., to be IN a relationship)
    - Orientation metaphor: metaphors that involve spatial relations such as UP-DOWN (e.g., feeling high/down)
    - Structural metaphor: metaphor in which an abstract concept (the target domain) is defined and expressed in terms of another conceptual domain (the source domain). Via conceptual mapping, target domain is understood by means of the structure of the source domain (e.g., LOVE IS A JOURNEY).
  - Target domain: For the present study, the target domains EMOTION and BRAIN/MIND/COGNITION were of particular interest (see Goschler, 2012 for classification of target domains).
  - Source domain: the various source domains of structural metaphors were classified as follows:
    - possession
    - parts of a whole
    - physical power
    - pressure, weight
    - journey, path
    - distance, proximity
    - physiological states, e.g., illness
    - visual perception, color
    - sensory perceptions, e.g., taste, temperature, auditory perception
    - other

In addition, the valence of all metaphorical utterances was coded as positive, negative, or neutral.

If an expression contained a metaphor and a literal reference to a specific internal state at the same time (e.g., “he is boiling with anger”), both the metaphorical expression (“boiling with…”) and the emotion term (“anger”) were coded.

Reliability: For the classification of IST, two raters coded the whole dataset independently. Interrater agreement was 91%. All cases of disagreement were solved by discussion. In addition, 11 independent raters assigned all IST to a valence category (positive, negative or neutral) and the dominant response was coded. The coding of metaphors depends on profound knowledge of cognitive metaphor theory. Therefore, a group of four trained linguists discussed the assignment of all metaphorical utterances to the type of metaphor, to the source and target domains, and to a valence category until agreement was achieved.

(3) **Formal thought disorder assessment:** The “Thought and Language Index” (TLI, Liddle et al., 2002) was used to assess formal thought disorder symptoms. The rating scale was developed and validated for the Thematic Apperception Task pictures and was found to have good psychometric properties. In addition, the TLI allows for a differentiation between FTD dimensions, according to the symptom clusters: impoverishment, disorganization, or dysregulation. A rater training was performed prior to the experiment. Two independent experienced senior psychiatrists evaluated the presence and the severity of formal thought disorder symptoms according to the TLI criteria. Rating results were discussed until high agreement was achieved. Afterwards, three transcripts were independently rated and interrater reliability was determined. Agreement of 80% was achieved. The blinded raters neither collected the data nor transcribed the audio files. The evaluation of TLI symptoms was carried out on the basis of pseudorandomized transcripts. Further information and a detailed description on the rating and classification procedure is given in Liddle et al. (2002).
  Scorings encompassed the following criteria: poverty of speech, weakening of goal, peculiar words, peculiar sentences, peculiar logic, perseveration, and distractibility. Items were grouped together to the main FTD dimensions suggested by Liddle and colleagues: impoverished thought/language, disorganized thought/language, and non-specific dysregulation.

### Results

In the proverb and metaphor interpretation test (Barth and Küfferle, 2001) groups significantly differed with respect to accuracy as healthy controls performed significantly better (Healthy Controls: *M* = 13.47; *SD* = 0.88; Patients with Depression: *M* = 12.16; *SD* = 2.68; *U* = −2.34, *p* < 0.02).

Altogether, 4831 literal terms or phrases for internal states (IST, 2221 produced by the clinical group and 2610 produced by the control group) and 1373 metaphorical expressions for internal states (ISM, 659 produced by the clinical group and 714 produced by the control group) were identified and coded. IST and ISM sum up to the amount of (figurative or non-figurative) ISL. Since the total number of word tokens used to describe the seven pictures differed between the participants, the number of IST and ISM and ISL was calculated relative to the total number of tokens. Absolute numbers and percentages were first computed for each participant, then averaged for group comparisons.

#### Amount, composition, and valence of internal state language

The control group was more talkative as compared to the clinical group, i.e., the healthy participants produced a significantly higher total number of words in order to describe the pictures. Accordingly, the patients with depression used fewer IST and ISM, which was significant for the IST (see Table 2). However, the proportions of IST, ISM, and ISL did not differ between the groups: terms that literally refer to internal states amounted to roughly 3% of all tokens in both groups. Metaphors for internal states made up nearly 1% of all tokens. The composition of ISL as a whole did also not reveal group differences: IST, i.e., literal expressions of internal states, took nearly 80%, and figurative expressions around 20%.

**Table 2:** Group comparisons of the amount and composition of ISL in the elicited speech production task: absolute numbers and proportions.

	**Patients with depression**		**Healthy controls**		**Group comparison**	
	**Mean**	***SD***	**Mean**	***SD***	***T***	**Sign**.
Total number of tokens	1645.41	551.71	2172.69	568.34	4.19	p < 0.001
Number of IST	50.48	28.35	72.50	39.73	2.79	p < 0.01
Number of ISM	14.98	9.79	19.83	14.29	–	ns
Number of ISL (IST+ISM)	65.45	35.52	92.33	49.95	2.71	p < 0.01
Proportion IST/tokens	3.1%	1.36	3.3%	1.5	–	ns
Proportion ISM/tokens	0.92%	0.57	0.90%	0.61	–	ns
Proportion ISL/tokens	4.02%	1.68	4.20%	1.87	–	ns
Proportion IST/ISL	77.26%	10.44	78.72%	10.44	–	ns
Proportion ISM/ISL	22.73%	10.44	21.27%	10.44	–	ns

The valence of the expressions of internal states was distributed as can be seen in Table 3. Patients with depression expressed fewer positive states as compared to healthy controls, which was significant for IST and led to a tendency for ISL.

**Table 3:** Valence of Internal State Language produced in the elicited speech production task: group comparisons.

		**Patients with depression**		**Healthy controls**		**Group comparison**	
		**Mean(%)**	**SD**	**Mean(%)**	**SD**		
IST	Positive	18.65	9.25	22.82	8.22	T(78) = 2.11	*P* < 0.04
	Negative	38.17	9.55	37.07	9.61	ns	
	Neutral	43.18	12.95	40.03	10.03	ns	
ISM	Positive	13.43	18.05	15,75	10.53	ns	
	Negative	22.17	17.03	21.79	14.06	ns	
	Neutral	62.37	24.94	62.46	19.82	ns	
ISL total	Positive	17.44	8.97	21.09	7.72	T(78) = 1.93	p = 0.058
	Negative	34.51	10.39	33.38	9.27	ns	
	Neutral	77.06	13.79	45.50	11.54	ns	

#### Analysis of internal state terms

**Figure 1:**
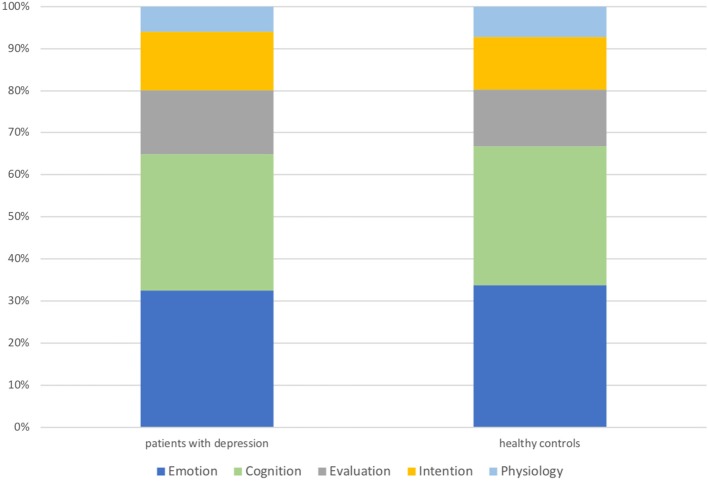
Composition of produced IST in the elicited speech production task: group comparisons.

Figure 1 shows that the composition of IST was very similar in both groups. There were no significant differences with respect to the proportion of the five IST categories relative to all IST or relative to all tokens.

#### Analysis of internal state metaphors

**Figure 2:**
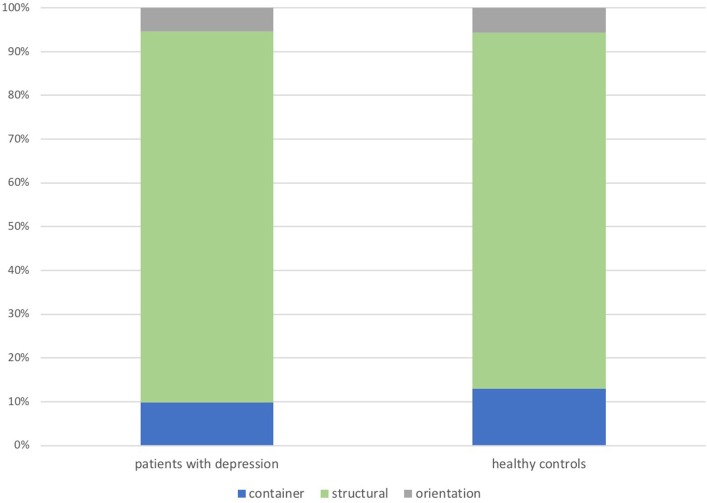
Distribution of produced metaphor types in the elicited speech production task: group comparisons.

Forty percent of metaphorical expressions for internal states referred to the target domain EMOTION and around 60% to the target domain MIND/BRAIN/COGNITION, without significant group differences. The distribution of metaphor types also did not yield group differences, as Figure 2 illustrates.

**Figure 3:**
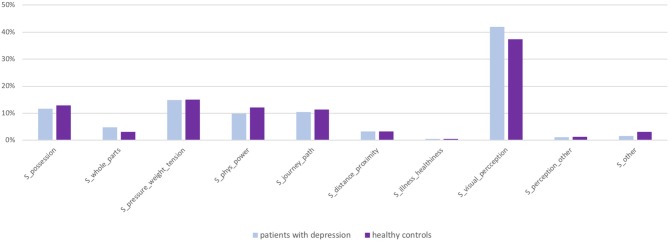
Distribution of source domains of structural metaphors in the elicited speech production task: group comparisons.

Figure 3 displays the distribution of the source domains of structural metaphors in both groups (relative to all structural metaphors), which again did not reveal group-specific differences. Visual perception was most commonly used as a source domain of structural metaphors.

#### Individual differences

Correlation analyses were conducted with the three TLI-dimensions and the proportion of words respective metaphors for internal states (relative to tokens). In addition to these broader categories, specific terms or metaphors for emotional states were included.

**Figure 4:**
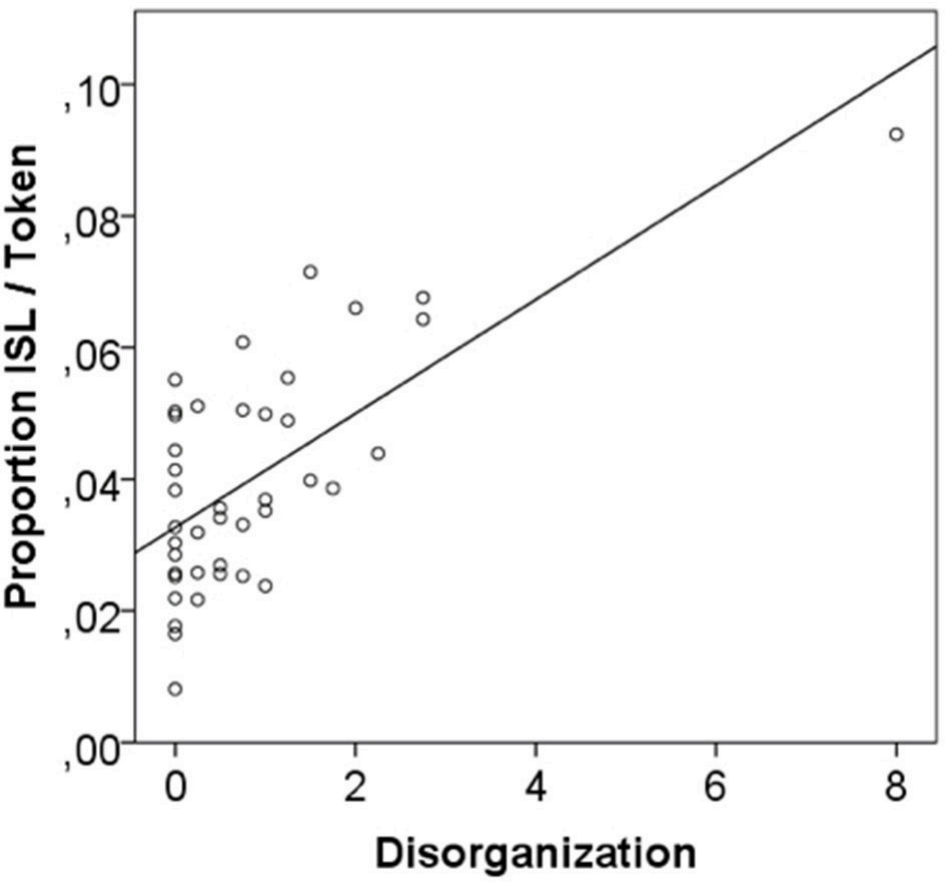
Correlation of patients' disorganization scores with their use of ISL in the elicited speech production task.

Table 4 shows that disorganized thought and language is positively associated with the verbalization of internal states: patients with high disorganization scores produced more words and metaphors for internal states in general (see Figure 4), as well as more metaphors for emotional states. The scales for dysregulation and (to a lesser degree) for impoverishment show a reverse relationship: impoverished speech as well as dysregulation symptomatology (e.g., perseveration) are related to a low amount of internal state language (significant for dysregulation). There were no significant correlations between valence categories of ISL and the TLI scores.

**Table 4:** Correlation analyses for the elicited speech production task: TLI-dimensions and proportion of internal state expressions.

	**Disorganization**	**Impoverishment**	**Dysregulation**
Proportion IST/tokens	0.46^***^	−0.08	−0.27
Proportion ISM/tokens	0.42^**^	−0.11	−0.46^***^
Proportion ISL/tokens	0.53^***^	−0.07	−0.39^*^
Proportion emotion terms/tokens	0.3	−0.04	−0.04
Proportion metaphors with target domain emotion/tokens	0.41^**^	−0.03	−0.07

* p < 0.05;

** p < 0.01;

*** *p < 0.005, N = 41. Values refer to Spearman's rho*.

##### Post-hoc correlations with proverb and metaphor interpretation results

No significant correlations were found for ISL relative to the number of tokens and performance in the proverb and metaphor interpretation test. The same holds for the different valence categories which were found to be unrelated to the idiom interpretation results. However, *post-hoc* analyses for TLI results revealed that the impoverishment dimension is significantly associated with performance in the proverb interpretation task. Patients with high impoverishment symptoms performed less accurate (Spearman's rho: 0.39, *p* < 0.05). No significant correlations were found for the other two TLI dimensions and performance in the proverb interpretation test.

### Summary of experiment 2

Overall, patients with depression produced a lower amount of speech when describing the pictures, supporting the “emptiness” or “poverty of speech” FTD phenomenon frequently observed during the assessment of spontaneous speech in clinical interviews (Nagels et al., 2016). The detailed analysis of the verbalization of internal states in elicited speech production demonstrated that patients with depression did not differ from healthy controls regarding the use of internal state terms and metaphorical expressions. In order to express emotional or cognitive processes metaphorically, both groups chose the same source domains. In addition, patients with depression verbalized positive content to a significantly smaller degree. On a group level, results did not support the assumption of differences between patients with depression and controls, except for the patients' tendency to disregard positive aspects more than controls. However, on an individual level, positive FTD symptoms (i.e., disorganization symptoms according to the TLI terminology) were found being associated with the amount of internal state language (figurative and literal). Patients with disorganized thought and language symptoms seem to focus strongly on internal states and processes or alternatively, reporting internal states leads to a disorganized speech pattern.

## Discussion

The aim of the present study was to investigate whether patients with depression are characterized by an altered processing of figurative language. To this end, we conducted two experiments with two different comprehensive samples. While the first task involved literal vs. figurative sentence completion, the second targeted elicited speech production. Results from both modalities point into the same direction: Patients with depression do not seem to understand and use figurative expressions differently from healthy controls. So far, it has been unclear if concretistic tendencies—which have been well documented in particular for schizophrenia—are also present in depression. Similar to Bartczak and Bokus (2015), the sentence completion data of the present study do not support a concretistic bias in the patients' choice of literal vs. figurative verbal expressions of internal states. Patients showed a similar pattern of sentence completion (Experiment 1) and the types and source domains of their own metaphorical expressions did not differ from those used by the controls (Experiment 2). Bartczak and Bokus (2015) explain the finding that subjects with depression do not have problems with processing metaphorical content in the light of cognitive metaphor theory: Metaphorical processing as a characteristic and natural way of human thinking occurs automatically, unconsciously, and effortlessly and does not require more resources than the processing of literal statements. Lakoff (2014) emphasizes that conceptual metaphors are an integral part of everyday reasoning. As pointed out in the introduction, figurative language serves to make abstract content more salient and enhances emotional involvement. Both functions of metaphoricity seem to be assessable for subjects with and without depression.

This finding, however, contrasts with the fact that patients with depression scored significantly lower in the proverb interpretation test (Barth and Küfferle, 2001) in both of our experiments. This might be attributed to task requirements and context. The experimental tasks allowed the participants to choose their preferred way of expressing internal states, whereby literal or figurative means are equally adequate. By contrast, the proverb interpretation test requires forced choice between alternatives, where only one interpretation is correct. Neither the sentence completion task nor the speech elicitation task were designed to assess concretistic thinking directly. The present results suggest that interpreting the abstract meaning of idioms and traditional sayings according to predetermined answers differs from choosing a figurative or non-figurative construction for emotional or other internal states.

The only group difference found in our study concerns the valence of ISL expressions. Patients with depression were found to verbalize positive states to a lesser extent as compared to controls. This finding is in line with the negative bias that has been shown to be distinctive for depression (e.g., Canli et al., 2004; Rude et al., 2004; Schlipf et al., 2013). The fact that the proportion of negative verbalizations did not differ between groups is consistent with Schlipf et al. (2013) who found impairments of positive, but not negative emotion processing in depressive patients compared to healthy controls. Both results confirm that attenuation of positive emotions seems to be one major characteristic of depressive patients (see also Clark and Watson, 1991). However, lack of group differences for negative valence category may also be attributed to the stimulus material triggering more negative than positive projections and depressing associations. It may be assumed that a balanced stimulus material consisting of both positive and negative social interactions and situations might have resulted in additional between-group differences with respect to the verbalizations' valence.

Beyond the pattern of differences and similarities between groups, our results uncovered associations between ISL and formal thought disorder. A disorganized speech pattern according to the TLI was associated with a pronounced use of internal state language in the patient group. It can be assumed that when talking about emotions or inner states verbal coherence tends to get rather loose (“tenuous or absent, or extraneous ideas intrude into the train of thought,” Liddle et al., 2002, p. 5) and/or non-logical reasoning (“conclusions are reached based on inadequate evidence or faulty logic,” Liddle et al., 2002, p. 5) arises. Interestingly, patients with high disorganization scores in the TLI (indicated by peculiar word use and peculiar sentence constructions) produced a significantly higher proportion of metaphors with emotion as target domain. Thus, disorganization may also promote the use of figurative expressions for emotional states as an attempt to clarify the message. The following utterance of a depressive patient illustrates sentence reformulations and metaphorical constructions (“duel,” conceptual metaphor ARGUMENT IS WAR) when the speaker tries to convey the intended meaning.

*(1) Könnt gut sein, dass da Mann und Frau sind, die sich da im Duell—ein Gespräch führen. So im Streitgespräch oder im Duell*.

It might be a man and a woman who are in a duel—have a conversation. Like in an argument or duel.

No association between TLI scores and valence was found, giving rise to the assumption that internal state language *per se* goes along with disorganized symptoms. In sum, verbalization of emotional content seems to increase the less organized the patients' thoughts and utterances are. In addition to the positive correlation between disorganization scores and ISL, a negative correlation was found with the TLI dysregulation scores. Dysregulation scores shaped by perseveration and distractability correlate negatively with the proportion of metaphors used, i.e., if the patients display high distractability or perseveration they use less metaphors when talking about internal states. Thus, dysregulation seems to weaken verbal creativity. The different directions of correlations between ISL and TLI scores support the conclusion of Liddle et al. (2002) that the scales for disorganization, impoverishment, and dysregulation reflect independent phenomena.

Finally, a positive correlation between performance in the proverb and metaphor interpretation test (Barth and Küfferle, 2001) and the impoverishment FTD dimension was found, which is in line with previous results. Kircher et al. (2014) also found that poverty of speech, slowed thinking, and concretism together form one “objective negative” factor on the TALD (Thought and Language Disorder) scale, a newly introduced nosologically-open clinical instrument. This negative TALD factor is highly associated with the TLI impoverishment items also encompassing *poverty of speech* as well as *weakening of goal* phenomena.

Taken together, the present study demonstrates that patients with depression as a group do not differ from controls when expressing internal states by literal and figurative verbal means, except for the tendency to pay attenuated attention to positive content. Moreover, the study revealed that the way patients verbalize internal states is related to the presence and form of formal thought disorders. This result is clearly illustrated by a deeper look into the transcript of a patient with depression, who received the highest scores for disorganization in the patients' group (see outlier in Figure 4 and transcript 2). The speaker describes a picture extremely negatively, using negative internal state terms as well as metaphors. Her speech contains reformulations, signs of derailment, and perseverations of the term “despair.” In addition, she attributes the difficult situation on the picture to her own life and her own negative feelings and anticipates events even worse than the depicted situation (death of own children). In contrast, a participant without depression (transcript 3), who describes the same picture, also recognizes the character's problem, but suggests a positive solution.

(2) *Tja…Da seh ich eine Frau die erschöpft… oder perspektivlos oder betrunken, wahrscheinlich mehr erschöpft… an ner Bank lehnt. Oder…auf der Bank hängt. Die verzweifelt ist … müde oder verzweifelt. Ich würde eher sagen der Haltung nach verzweifelt. Ja, entweder ist irgendwas passiert in meinem, in ihrem Leben, das sie nicht so schnell verkraften kann. Was will ich gar nicht sagen, weil das wär… aber es muss halt einiges passiert sein, was, was sie, dazu antreibt sich so erschöpft und mutlos dahin zu hängen. In dem Bild sehe ich nur Verzweiflung. Vielleicht hat sie Kinder, denen irgendwas passiert ist. Vielleicht sind die Eltern, dass irgendwas, also was Böses passiert ist, dass sie jemanden verloren hat und hilflos ist und verzweifelt und nicht mehr weiß, wie's weitergehen soll, ne. Weil das mit den Kindern wollte ich eigentlich gar nicht ansprechen, nee, weil da denk ich nicht drüber nach. Nee… weil, meine Kinder sollen nach mir gehen, so was wünsch ich keinem*. Well… I see a woman leaning against a bench, exhausted or without perspective or drunk, probably more exhausted. Or hanging on the bench. Who is desperate… tired or desperate. I would say desperate according to her posture. Yes, something has happened in my, in her life, that she cannot cope with. I don't want to say what because that would be… But something happened that drives her hanging there so exhausted and so discouraged. I see only despair in this picture. Perhaps she has children and something happened to them. Perhaps it's something with her parents, that something, something bad, happened to them, that she has lost someone and is helpless and desperate and does not know how to go on. Because—I didn't want to mention the thing with the children because I don't want to think about that. No. Because … my children should go after me; I don't wish anything like this to anyone.

*(3) das Bild strahlt ähm, Trauer aus und ähm, Einsamkeit und hm, Abkehr. Also die ganze Haltung drückt Abkehr aus und ähm Hoffnungslosigkeit auch irgendwo. Hm, vorher gab es vermutlich einen Streit oder eine schlechte Nachricht, ähm, stattgefunden. Deswegen ein Zusammenbruch erfolgt ist und deswegen die Frau sehr traurig und resigniert ist und nicht mehr weiter weiß. Ich hoffe sie rappelt sich auf und beschließt, ähm, aus der Situation etwas Positives zu machen und sie vielleicht jemanden anruft und mit demjenigen die Situation bespricht oder sich erst mal was zu essen macht und einen heißen Kakao und dann weiter sieht*.

The picture radiates sadness and loneliness and “turning away.” The whole posture expresses “turning away” and hopelessness too. Hm, there must have been a dispute or bad news. Which caused a breakdown and caused that the woman is very sad and resigned and does not know how to go on. I hope she gets on her feet again and decides to make something positive out of the situation and that she perhaps calls someone to talk about the situation or that she gets some food and a hot chocolate and then considers what to do next.

To conclude, the results of present study broaden the understanding of figurative processing in depression: patients with depression do not show a concretistic bias regarding their choice of literal vs. figurative verbal expressions of internal states. However, their internal state language is modified by the presence of specific symptoms of formal thought disorders.

## Ethics statement

This study was carried out in accordance with the recommendations of the Ethics Committee of the medical faculty of the University of Marburg. The protocol was approved by the Ethics Committee of the medical faculty of the University of Marburg. All subjects gave written informed consent in accordance with the Declaration of Helsinki.

## Author contributions

CK, AN, TK, NM acquisition, analysis, and interpretation of data for the work. CK, AN, TK drafting the work and revising it critically for important intellectual content. CK and AN agreement to be accountable for all aspects of the work in ensuring that questions related to the accuracy or integrity of any part of the work are appropriately investigated and resolved.

### Conflict of interest statement

The authors declare that the research was conducted in the absence of any commercial or financial relationships that could be construed as a potential conflict of interest.
